# Community organizing and community health: piloting an innovative approach to community engagement applied to an early intervention project in south London

**DOI:** 10.1093/pubmed/fdv017

**Published:** 2015-02-26

**Authors:** Matthew Bolton, Imogen Moore, Ana Ferreira, Crispin Day, Derek Bolton

**Affiliations:** 1 Citizens UK, London E1 2JA, UK; 2 Institute of Psychiatry, Psychology and Neuroscience, King's College London, London SE5 8AF, UK

**Keywords:** communities, social determinants

## Abstract

**Background:**

The importance of community engagement in health is widely recognized, and key themes in UK National Institute for Health and Clinical Excellence (NICE) recommendations for enhancing community engagement are co-production and community control. This study reports an innovative approach to community engagement using the community-organizing methodology, applied in an intervention of social support to increase social capital, reduce stress and improve well-being in mothers who were pregnant and/or with infants aged 0–2 years.

**Methods:**

Professional community organizers in Citizens-UK worked with local member civic institutions in south London to facilitate social support to a group of 15 new mothers. Acceptability of the programme, adherence to principles of co-production and community control, and changes in the outcomes of interest were assessed quantitatively in a quasi-experimental design.

**Results:**

The programme was found to be feasible and acceptable to participating mothers, and perceived by them to involve co-production and community control. There were no detected changes in subjective well-being, but there were important reductions in distress on a standard self-report measure (GHQ-12). There were increases in social capital of a circumscribed kind associated with the project.

**Conclusions:**

Community organizing provides a promising model and method of facilitating community engagement in health.

## Introduction

The importance of communities being involved in their own health is widely recognized, and the UK National Institute for Health and Clinical Excellence (NICE) in its public health guidance makes specific recommendations for enhancing community engagement, key themes in which are co-production and community control.^1^ A recent systematic review of community engagement interventions^2^ concludes that they are effective across a wide range of contexts, while noting at the same time the limited evidence base, especially in the UK. One of the many kinds of health issues to which community involvement is relevant is child development. The Marmot Review of Health Inequalities in England^3^ has as its Policy Objective A: ‘Give every child the best start in life’. The UK Chief Medical Officer's recent report^4^ identifies maternal psychosocial stress during pregnancy and baby's infancy as a risk factor for healthy development and its recommendations include strengthening social support networks.

We report here a project carried out in south London during 2013 that addressed issues of community engagement and co-production in relation to social support for new mothers. The project involved collaboration between Citizens-UK and King's Health Partners. King's Health Partners^5^ is an Academic Health Sciences Centre in south London committed to improving local public health.^6^ Citizens-UK^7^ is the largest community-organizing charity in the UK. It uses the ‘broad-based’ community–organizing model and methodology, which is well theorized, deriving from the work of Saul Alinsky in Chicago,^8,9^ and which is applied by community organizations throughout the USA and by Citizens-UK in the UK. Key features of the approach include building trust-based reciprocal relationships among individuals in already existing communities, particularly civic institutions, fostering networks among diverse institutions, developing community leadership and working towards goals decided by communities. Citizens-UK employs paid, trained professional community organizers who work with volunteer community leaders. In recent years, its work includes campaigning for a Living Wage, improving the treatment of people seeking asylum, and enabling young people to work with shopkeepers and the police to make streets safer. London Citizens is part of Citizens-UK and has 210 civic institutions as dues-paying members, largely faith-based and education organizations. There are 10 London Citizens member communities situated in and around the area of south London in which this project is based. The broad-based community-organizing approach is well suited to optimize community engagement using principles of co-production and community leadership as recommended by NICE, as noted above.^1^ Since Marmot's Review of health inequalities,^3^ Citizens-UK and its member institutions increasingly recognize that many of their socio-political concerns—for example community cohesiveness, the living wage and safer streets—are related to the ‘wider social determinants of health’. The current project is one of several in which Citizens-UK and its member institutions are working with health services and commissioners on community health.

The broad aim of this collaboration between Citizens-UK and King's Health Partners is to make use of social capital in existing civic institutions and to combine this with clinical academic resources to translate and evaluate early prevention science for local mothers and babies. Social capital was defined by Putnam^10^ as the quality of participation in formal civic organizations, informal social networks and voluntary associations, subsequent authors including also the resources that these social connections bring,^11^ and the concept has been applied specifically to mothers and their influence on children's well-being.^12^ The present study was based on a conceptual model linking increase of social support and social capital to reduction of stress. The specific aim was to evaluate a community-led intervention of social support to increase social capital, reduce stress and improve well-being in mothers who were pregnant and/or with infants aged 0–2 years. The project has the working title ‘Strengthening babies' futures through evidence-based community action’ and addressed the following four specific research questions:

Question 1: Is it feasible to devise and implement a community-led, community-level intervention providing social support for mothers who are pregnant or with young children?
Question RQ2: Is the intervention acceptable to the mothers taking part?
Question RQ3: Is the approach we used—involving community organizing and health expertise—consistent with NICE (2008) guidance on community engagement^1^ in that it involves co-production and community control? And if so, were such factors seen by participating mothers as facilitating engagement?
Question RQ4: Does the intervention show signs of having the intended beneficial effects on mothers' social capital, stress and subjective well-being?

## Methods

In the early stages of the project, senior staff of London Citizens, part of Citizens-UK, had meetings with leaders of one member institution in south London, a Baptist church on the border of the boroughs of Lambeth and Southwark, to discuss and agree on the broad aims of the then proposed project. The prior determined intervention, agreed in broad terms with clinical academic psychologists in King's Health Partners, was only that interested members of the church and local London Citizens professional community organizers would work together to facilitate forming a local new mothers’ group with the aim of increasing social support. The process from that point was left to the church community and the Community Organizers, and the subsequent process and outcomes that emerged, including ‘the intervention’, will accordingly be described in the Results section.

### Measures

To address Research Question 1, we took records of numbers, meetings and activities. To address Research Question 2, we used the Social Support Programme Acceptability Rating Scale, an adaptation of the Treatment Acceptability Rating Scale,^13^ comprising ratings of user satisfaction for seven different aspects of the programme of social support using a four-point Likert scale. (A copy of the scale used is available as Supplementary data.) We approached Research Question 3—on the perceived extent and effectiveness of co-production and joint control—by constructing a questionnaire, ‘What helped and what didn't in the project’, derived from applicable NICE recommendations on community engagement,^1^ specifically from those related to co-production and community control. The constructed questionnaire has two parts. Part (A) comprises 11 questions with responses on a three-point Likert scale: (1) hardly at all/no; (2) yes somewhat; (3) yes a lot; plus an additional response option: ‘not clear/do not know’ (not scored). If the respondent answers (2) or (3), they are asked to respond to a second part (B) of the question: ‘Has this helped people be involved in the project?’—responding on the same three-point Likert scale. (A copy of the scale used is available as Supplementary data.)

To address Research Question 4, we used the following measures:

- The General Health Questionnaire-12 (GHQ-12),^14^ a 12-item self-report questionnaire in standard use. We used the Likert scoring method that results in a score ranging from 0 to 36, classified for interpretation into five categories.^15^
- The Warwick-Edinburgh Mental Well-being Scale (WEMWBS),^16^ a 14-item self-report questionnaire answered using a five-point Likert scale of frequency.
- The Adapted Social Capital Questionnaire, an adaptation of the World Bank's Social Capital Integrated Questionnaire,^17^ omitting sections not applicable to this project, administered as a structured interview. (A copy of the adapted questionnaire used is available as Supplementary data, supporting information, Supporting methods and Adapted Social Capital Questionnaire.)

### Planned analytic strategy

For the GHQ-12, WEMWBS and Adapted Social Capital Questionnaire, we planned to examine descriptive statistics and test for pre-/post statistical significance using the paired sample *t*-statistic using SPSS-19.^18^ In addition to computing conventional statistical significance, we planned to estimate the size of effects as 95% confidence intervals for mean differences.^19^ In addition, for the GHQ-12 and WEMWBS, we used a repeated measures single-case methodology allowing statistical analysis on multiple data points and computation of intervention effect sizes and *P*-values; we planned to use the Tau-U statistic, derived from Kendall's rank correlation and the Mann–Whitney *U* between groups test,^20^ software for which is available free on the web.^21^ This typically requires ∼8 minimum data points in each of the baseline and intervention phases. We used the repeated measures single-case methodology because of its valuable sensitivity to individual differences and also to avoid failure to collect second data points, which threatens the internal validity of simple two-measure pre–post designs, following in this respect the methodology used, for example, by the UK Increasing Access to Psychological Therapies (IAPT) programme.^22^

Shopping vouchers were offered to participants in compensation for their time taken in the evaluation (not for participation in the programme itself). For mothers participating in the research, we collected basic demographic information including self-declared ethnicity using standard UK classifications, from the UK 2011 Census.^23^

The evaluation protocol was reviewed by the King's College London Research Ethics Sub-Committee, ref. PNM 12/13-85. Written informed consent was obtained from participants in the evaluation.

## Results

### The community-led process and outcomes

As noted above, the prior determined intervention was only that interested members of the church and the London Citizens community organizers would work to facilitate forming a local mothers' social support group. Several key features of the process that emerged from that starting point included:

1. A team of ∼5 or 6 volunteer community leaders and two part-time professional Community Organizers from London Citizens (total 0.8 full-time equivalent) helped make links with other local institutions, drawing partly on previous existing relationships. The participating institutions grew rapidly to include three churches, one Islamic Centre, one faith-based charity that ran a large mother and toddler group, one after-school project and one youth club.
2. The team of local community leaders, which was expanded as above, and two part-time professional Community Organizers also worked together to seek out mothers, pregnant or with children under 2 years, and invited them to take part. They approached mothers in participating organizations but also made contact with mothers outside these organizations, by door knocking on local estates and in public spaces such as parks. They also gave public talks and then followed up with interested individuals. Some mothers were signposted to the programme by a friend or community leader/teacher/professional. Approximately 25 women were asked and wanted to participate to varying extents.
3. What emerged once the mothers began to meet was that they formed their own social support network. They planned regular meetings, initially spaced out by a week or two, then weekly. In advance of their meetings the participating mothers agreed on relevant topics to discuss such as breast feeding, sleep routines, relationships with partners, managing stress, housing and juggling work and child care. The women also made requests to the organizations involved, which were positively responded to, such as requests to the participating civic institutions for rooms and facilities for meeting, and to health providers for educational classes on parenting, diet and child development, as well as information talks from early years providers.

In summary, the community-led ‘intervention’ that evolved comprised mothers meeting together to provide mutual social support, choosing for discussion topics, concerns and worries, and sharing advice, these sessions being supplemented by requested health information and educational workshops. In total there were 23 meetings of participating mothers, of 2.5 h duration, 6 of which were workshops as above. There were also two lunchtime events, including community leaders and health professionals as well as mothers, to review progress and plans, and 6 meetings on evaluating the project including on average 5 participating mothers who wanted to be involved in its design and implementation.

### Evaluation process and results

For the purposes of evaluating the project and addressing the research questions, we aimed to gather data from a convenience sample of 15 mothers among those participating in the project; when the first 15 agreed to take part in the evaluation, recruitment was ended. Baseline data were collected during a gap of 6–8 weeks prior to full commencement of the programme. Data were gathered by the Community Organizers and one of the participants in the projects using the model of ‘action research’^24,25^ and specifically ‘community-based participatory research’,^26^ in which academic researchers and participants work together to investigate the research questions.

#### The evaluation sample

The 15 mothers participating in the evaluation had a mean age of 31 years (range 19–40) and the main self-declared ethnicities were ‘African’ (8/15) and ‘white’ (3/15). Baseline GHQ-12-assessed levels of distress showed heterogeneity and relatively high levels: 47% (7/15) of the participating mothers had scores in the ‘distressed’ or ‘severely distressed’ ranges, and 27% (4/15) were in the highest, ‘severely distressed’ category.

Findings related to the Research Questions were as follows. Firstly, it was found to be feasible to devise and implement a community-led, community-level intervention providing social support for new mothers. Secondly, the intervention was found to be acceptable: mean scores on the Social Support Programme Acceptability Rating Scale are given in Table 1:

**Table 1:** Mean scores on the Social Support Programme Acceptability Rating Scale

*Item*	*Mean score*
Did you feel involved in helping to plan what social support you would find helpful?	2.13
Did you feel able to make changes to the plan to suit your needs during the programme?	1.80
Was the planned social support actually provided?	2.40
Did you like the way the programme was provided to you?	2.67
Did you like the members of the community who were providing the support?	2.73
On balance, did you find that the programme made life better for you?	2.67
In an overall, general sense, how satisfied are you with the programme?	2.47

Four-point Likert scale: 0 = not at all; 1 = a little: 2 = quite a lot; 3 = a great deal.

It can be seen in Table 1 that the programme of social support was acceptable to participating mothers across a broad range of indices.

On the third research question, regarding the extent and effectiveness of perceived co-production and community control, group mean scores for the two parts of the ‘What helped and what didn't in the project’ questionnaire are given in Table 2.

**Table 2:** Mean scores on the Community Co-Production Scale

*Item*		*Part A*	*Part B*
1	Have you helped identify what needs to be done and how to do it?	2.57	2.69
2	Have you felt that your views have been taken into account?	2.73	2.73
3	Has local diversity been taken into account appropriately (such as where people live, faith, ethnic background)?	3.00	3.00
4	Have plans about how to go about the project been agreed jointly with you?	2.80	2.92
5	Has the project used existing community networks (such as churches, mosques, play-groups)?	2.80	2.93
6	Has the project provided the structures and resources needed for you to participate?	2.80	2.87
7	Has the project involved people who may have otherwise felt not part of social groups?	2.79	2.93
8	Have London Citizens staff (named) helped to organize the project?	3.00	2.93
9	Have they explained the importance of the project for health?	2.93	2.80
10	Has the project built relationships between local institutions?	2.50	2.64
11	Have you felt more able to make relationships with people in different organizations?	2.73	2.80

Three-point Likert scale: 1 = hardly at all/no; 2 = yes somewhat; 3 = yes a lot; plus additional option: ‘not clear/do not know’ (not scored).

It can be seen in Table 2 that the 11 items as to whether the project incorporated features related to co-production and community control were generally positively endorsed by the participating mothers in Part A, and further, in Part B, for each of the 11 items, the secondary question whether that particular feature helped people be involved in the project was also positively endorsed, consistent with the principle of the NICE model that enhancing co-production and control promotes community engagement.

Related to the fourth research question, Table 3 shows descriptive statistics for GHQ-12-assessed distress pre-/post- social support intervention (using first and last data points collected) and the result of a paired-samples *t*-test.

**Table 3:** Descriptive statistics and the result of a paired-samples *t*-test for GHQ-12 scores by social support intervention

	*Before social support intervention*		*After social support intervention*		**n**	*95% CI for mean difference*	*t*	*df*	*P*
*Outcome*	*M*	*SD*	*M*	*SD*					
	15.20	7.60	10.27	5.51	15	−0.43, 10.30	1.97	14	>0.05

It can be seen in Table 3 that there was a reduction in the group mean of nearly 5 points, equating to approximately two-thirds of the pre-test standard deviation. The 95% confidence intervals of the reduction range between just below zero to ∼10, suggesting an important effect, though the range is large, consistent with a small sample size, and, with the lower limit below zero, the t- value is below the conventional 5% level of statistical significance.

Single-case repeated measures analyses of GHQ-12 scores using the TAU-U statistic showed that 20% of the sample (3/15) showed statistically significant reduction in the period of the intervention compared with baseline period scores.

The Warwick-Edinburgh Mental Well-being Scale (WEMWBS) showed no important and no statistically significant pre/post changes either for the group as a whole or for any of the individual participants.

Mean scores on the Adapted Social Capital Questionnaire showed statistically significant changes from baseline to outcome on two items: ‘how many days in the past 12 months did you participate in community activities?’ [paired *t* (14) = 2.49, *P* = 0.026], and ‘how well do people in your neighbourhood help each other out these days?’[paired *t* (14) = 3.29, *P* = 0.005]. However, there were no statistically significant changes on other items (such as those related to trust in officials or perceived political influence). In brief, there were positive findings for local social support, but no detected effects on broader social capital.

## Discussion

### Main findings of this study

The main findings of this study are that the broad-based model of community organizing that involves benefitting from social capital in civic institutions can be applied to a health-related intervention with measurable health outcomes, in this case a social support programme for new mothers, that this was acceptable to participants, and that the model as applied in the present study is broadly compliant with NICE guidance to facilitate community engagement by co-production and community control. Community control had the outcome that the original general idea of providing social support evolved to include other components, particularly health educational workshops. Finally, there were signs that the intervention had intended effects on some key outcomes of interest, specifically increases in social capital at least of a circumscribed kind associated with the project, and a decrease in GHQ-12-assessed levels of maternal distress.

### What is already known on this topic

A recent systematic review of community engagement interventions^2^ concludes that community engagement interventions are effective across a wide range of contexts, while noting at the same time the limited evidence base, especially in the UK, and the uncertainty about how communities might be best engaged. NICE Guidance on community engagement recognizes that ‘community champions’ are likely to be required for promotion and organization,^1^ but details are unspecified. Community organizing has been applied in community health-related interventions, mainly in the USA.^27^ Many community-based interventions lack attention to measurable health outcomes,^2^ including early prevention projects.^28^

### What this study adds

To our knowledge this is the first report of community organizing applied to health in the UK. Because community organizing is a transferable methodology, as is focus on health issues, the model and procedures used in this study are in principle reproducible in new locations. The study incorporates measurement of health outcomes using standardized measures in a quasi-experimental design, recommended in policy documents such as the Allen Report.^27^ It is also to our knowledge the first report to assess quantitatively adherence to and effectiveness of principles of co-production and community control as recommended by NICE.^1^ The study provides a promising basis for a larger-scale study of community organizing facilitated communities, working with universal maternity services, to provide social support and health education to women in pregnancy and post natal with a view to improving both maternal and baby outcomes.

### Limitations of this study

The measure of co-production and community control is newly constructed and has face validity only. The study is limited by a small sample size and requires replication with a larger population.

## Supplementary data

Supplementary data are available at *PUBMED* online.

## Funding

This work was supported by the Guy's & St Thomas’ Charity (EFT 120604); and the National Institute for Health Research (NIHR) Biomedical Research Centre at South London and Maudsley NHS Foundation Trust and King's College London, to D.B. This paper represents independent research part funded by the National Institute for Health Research (NIHR) Biomedical Research Centre at South London and Maudsley NHS Foundation Trust and King's College London. The views expressed are those of the authors and not necessarily those of the NHS, the NIHR or the Department of Health.

## Supplementary Material

Supplementary Data
