# Supplementary material for: Community organizing and community health: piloting an innovative approach to community engagement applied to an early intervention project in south London
Source: J Public Health (Oxf). 2015 Feb 26;38(1):115–21. doi: 10.1093/pubmed/fdv017 (PMC4750521; doi:10.1093/pubmed/fdv017)
Supplement: Supplementary Data [file supp_38_1_115__index.html]

Community organizing and community health: piloting an innovative approach to community engagement applied to an early intervention project in south London — Community organizing and community health: piloting an innovative approach to community engagement applied to an early intervention project in south London — Supplementary Data 

# Community organizing and community health: piloting an innovative approach to community engagement applied to an early intervention project in south London

## Supplementary Data

Supplementary Data

**Files in this Data Supplement:**

- Supplementary Data - Docx file
