# Supplementary material for: Community organizing and community health: piloting an innovative approach to community engagement applied to an early intervention project in south London
Source: J Public Health (Oxf). 2015 Feb 26;38(1):115–21. doi: 10.1093/pubmed/fdv017 (PMC4750521; doi:10.1093/pubmed/fdv017)
Supplement: Supplementary Data [file supp_fdv017_fdv017supp.docx]

Supporting Information

Supporting methods

Social Support Programme Acceptability Rating Scale

| **Social Support Programme Acceptability Rating Scale** |
| --- |

The following 10 questions focus on your impressions of the Social Support Programme.

**For each question, please circle the statement that best expresses your opinion.**

PLEASE CIRCLE ONE ANSWER.

**1. Did you feel involved in helping to plan what social support you would find helpful?**

| not at all | a little | quite a lot | a great deal |
| --- | --- | --- | --- |
| 0 | 1 | 2 | 3 |

**2. Did you feel able to make changes to the plan to suit your needs during the programme?**

| not at all | a little | quite a lot | a great deal |
| --- | --- | --- | --- |
| 0 | 1 | 2 | 3 |

**3. Was the planned social support actually provided?**

| not at all | a little | quite a lot | a great deal |
| --- | --- | --- | --- |
| 0 | 1 | 2 | 3 |

**4. Did you like the way the programme was provided to you?**

| not at all | a little | quite a lot | a great deal |
| --- | --- | --- | --- |
| 0 | 1 | 2 | 3 |

**5. Did you like the members of the community who were providing the support?**

| not at all | a little | quite a lot | a great deal |
| --- | --- | --- | --- |
| 0 | 1 | 2 | 3 |

**6. On balance, did you find that the programme made life better for you?**

| not at all | a little | quite a lot | a great deal |
| --- | --- | --- | --- |
| 0 | 1 | 2 | 3 |

**7. In an overall, general sense, how satisfied are you with the programme?**

| not at all | a little | quite a lot | a great deal |
| --- | --- | --- | --- |
| 0 | 1 | 2 | 3 |

“What helped and what didn’t in the project” questionnaire

| **What helped and what didn’t in the project** |
| --- |

These are questions about the project you have been taking part in to provide social support to mothers.

Circle the response that best applies to you. Each question has two parts, A and B. If you respond (2) or (3) to Part A of a question, please also answer Part B; otherwise go straight to the next question.

**1. Part A: Have you helped identify what needs to be done and how to do it?**

| Hardly at all / No | Yes, somewhat | Yes, a lot | Not clear / don’t know |
| --- | --- | --- | --- |
| 1 | 2 | 3 | 0 |

**1. Part B. (If 2 or 3) Do you think that this has helped people be involved in the project?**

| **Hardly at all / No** | **Yes, somewhat** | **Yes, a lot** | **Not clear / don’t know** |
| --- | --- | --- | --- |
| **1** | **2** | **3** | **0** |

**2. Part A: Have you felt that your views have been taken onto account?**

| Hardly at all / No | Yes, somewhat | Yes, a lot | Not clear / don’t know |
| --- | --- | --- | --- |
| 1 | 2 | 3 | 0 |

**2. Part B. (If 2 or 3) Do you think that this has helped people be involved in the project?**

| Hardly at all / No | Yes, somewhat | Yes, a lot | Not clear / don’t know |
| --- | --- | --- | --- |
| 1 | 2 | 3 | 0 |

**3. Part A: Has local diversity been taken into account appropriately (such as where people live, faith, ethnic backgrounds)?**

| Hardly at all / No | Yes, somewhat | Yes, a lot | Not clear / don’t know |
| --- | --- | --- | --- |
| 1 | 2 | 3 | 0 |

**3, Part B. (If 2 or 3) Do you think that this has helped people be involved in the project?**

| Hardly at all / No | Yes, somewhat | Yes, a lot | Not clear / don’t know |
| --- | --- | --- | --- |
| 1 | 2 | 3 | 0 |

**4. Part A: Have plans of what to do been agreed jointly with you?**

| Hardly at all / No | Yes, somewhat | Yes, a lot | Not clear / don’t know |
| --- | --- | --- | --- |
| 1 | 2 | 3 | 0 |

**4. Part B. (If 2 or 3) Do you think that this has helped people be involved in the project?**

| Hardly at all / No | Yes, somewhat | Yes, a lot | Not clear / don’t know |
| --- | --- | --- | --- |
| 1 | 2 | 3 | 0 |

**5. Part A: Has the project used existing community networks (such as churches, mosques, play-groups)?**

| Hardly at all / No | Yes, somewhat | Yes, a lot | Not clear / don’t know |
| --- | --- | --- | --- |
| 1 | 2 | 3 | 0 |

**5. Part B. (If 2 or 3) Do you think that this has helped people be involved in the project?**

| Hardly at all / No | Yes, somewhat | Yes, a lot | Not clear / don’t know |
| --- | --- | --- | --- |
| 1 | 2 | 3 | 0 |

**6. Part A: Has the project provided the structure and resources needed for you to participate? (Such as room, hospitality, crèches, classes)?**

| Hardly at all / No | Yes, somewhat | Yes, a lot | Not clear / don’t know |
| --- | --- | --- | --- |
| 1 | 2 | 3 | 0 |

**6. Part B. (If 2 or 3) Do you think that this has helped people be involved in the project?**

| Hardly at all / No | Yes, somewhat | Yes, a lot | Not clear / don’t know |
| --- | --- | --- | --- |
| 1 | 2 | 3 | 0 |

**7. Part A: Has the project involved people who may have otherwise felt no part of social groups?**

| Hardly at all / No | Yes, somewhat | Yes, a lot | Not clear / don’t know |
| --- | --- | --- | --- |
| 1 | 2 | 3 | 0 |

**7. Part B. (If 2 or 3) Do you think that this has helped people be involved in the project?**

| Hardly at all / No | Yes, somewhat | Yes, a lot | Not clear / don’t know |
| --- | --- | --- | --- |
| 1 | 2 | 3 | 0 |

**8. Part A: Have London Citizens staff (named) helped to organise the project?**

| Hardly at all / No | Yes, somewhat | Yes, a lot | Not clear / don’t know |
| --- | --- | --- | --- |
| 1 | 2 | 3 | 0 |

**8. Part B. (If 2 or 3) Do you think that this has helped people be involved in the project?**

| Hardly at all / No | Yes, somewhat | Yes, a lot | Not clear / don’t know |
| --- | --- | --- | --- |
| 1 | 2 | 3 | 0 |

**9. Part A: Have London Citizens staff (named) explained the importance of the project for health?**

| Hardly at all / No | Yes, somewhat | Yes, a lot | Not clear / don’t know |
| --- | --- | --- | --- |
| 1 | 2 | 3 | 0 |

**9. Part B. (If 2 or 3) Do you think that this has helped people be involved in the project?**

| Hardly at all / No | Yes, somewhat | Yes, a lot | Not clear / don’t know |
| --- | --- | --- | --- |
| 1 | 2 | 3 | 0 |

**10. Part A: Has the project helped build relationships between local institutions?**

| Hardly at all / No | Yes, somewhat | Yes, a lot | Not clear / don’t know |
| --- | --- | --- | --- |
| 1 | 2 | 3 | 0 |

**10. Part B. (If 2 or 3) Do you think that this has helped people be involved in the project?**

| Hardly at all / No | Yes, somewhat | Yes, a lot | Not clear / don’t know |
| --- | --- | --- | --- |
| 1 | 2 | 3 | 0 |

**11. Part A: Have you felt more able to make relationships with people in different organisations?**

| Hardly at all / No | Yes, somewhat | Yes, a lot | Not clear / don’t know |
| --- | --- | --- | --- |
| 1 | 2 | 3 | 0 |

**11. Part B. (If 2 or 3) Do you think that this has helped people be involved in the project?**

| Hardly at all / No | Yes, somewhat | Yes, a lot | Not clear / don’t know |
| --- | --- | --- | --- |
| 1 | 2 | 3 | 0 |

Adapted Social Capital Questionnaire

**Adapted Social Capital Questionnaire**

“The following questions are to find out about your social networks.”

**1. Groups and Networks**

1. I’d like to start by asking you about the groups or organizations, networks, associations to which you belong. These could be formally organized groups or just groups of people who get together regularly to do an activity or talk about things.

Please detail below:

(e.g. community, religious, health, youth, sports groups)

|  |
| --- |

1. Compared to when this project started*, do you participate in more or fewer groups or organizations? (*Asked at end of project.)

1 More

2 Same number

1. Fewer

**Now the following questions are about your involvement in the Social Support Programme based at [name of several organisations that hosted meetings] OR the main group you are part of __________________________**

1. How many times in the past 12 months did you participate in this group’s activities, e.g. by attending meetings or doing group work?
2. When there is a decision to be made in the group, how does this usually come about?

1 Decision is imposed from outside

2 The leader decides and informs the other group members

3 The leader asks group members what they think and then decides

4 The group members hold a discussion and decide together

5 Other (specify __________________________________________)

1. Does this group work or interact with other groups with similar goals in the neighborhood?

1 No

2 Yes, occasionally

3 Yes, frequently

1. What is the most important source of expertise or advice which this group receives?

1 From within the membership

2 From other sources within the community

3 From sources outside the community

Networks

1. About how many close friends do you have these days? These are people you feel at ease with, can talk to about private matters, or call on for help.

**The following questions are more broader about the neighbourhood you live in.**

**2. Trust and Solidarity**

In every community, some people get along with others and trust each other, while other people do not. Now, I would like to talk to you about trust and solidarity in your neighborhood.

1. Now I want to ask you how much you trust different types of people. On a scale of 1 to 5, where 1 means a very small extent and 5 means a very great extent, how much do you trust the people in that category?

|  | 1. To a very small extent  2. To a small extent  3. Neither small nor great extent  4. To a great extent  5. To a very great extent |
| --- | --- |
| A. Local /central government officials |  |
| B. Nurses and doctors |  |

1. Do you think that prior to/ since the project*, the level of trust in this neighborhood has… (*depending on when this questionnaire is completed)

1 Gotten better

2 Gotten worse

3 Stayed about the same

1. How well do people in your neighborhood help each other out these days?

1 Always helping

2 Helping most of the time

3 Helping sometimes

4 Rarely helping

5 Never helping

**3. Collective Action and Cooperation**

1. In the past 12 months, have you worked with others in your neighborhood to do something for the benefit of the community?

| 1 | Yes | 2 | No |
| --- | --- | --- | --- |

1. Altogether, how many days in the past 12 months did you participate in community activities?
2. What were the three main such activities in the past 12 months? Was participation in these voluntary or required?

|  | Voluntary | Required |
| --- | --- | --- |
|  |  |  |
|  |  |  |
|  |  |  |

**5. Social Cohesion and Inclusion**

1. How strong is the feeling of togetherness or closeness in your neighborhood?

1 Very distant

2 Somewhat distant

3 Neither distant nor close

4 Somewhat close

5 Very close

1. There are often differences in characteristics between people living in the same neighborhood. For example, differences in wealth, income, social status, ethnic background, race. There can also be differences in religious or political beliefs, or there can be differences due to age or sex. To what extent do any such differences characterize your neighborhood?

1 To a very great extent

2 To a great extent

3 Neither great nor small extent

4 To a small extent

5 To a very small extent

Sociability

I am now going to ask a few questions about your everyday social interactions.

1. In the last month, how many times have you met with people in a

public place either to talk or to have food or drinks?

1. In the last month, how many times have people visited you in your home?
2. In the last month, how many times have you visited people in their home?
3. In the last three months, how many times have you gotten together with

people to play games, sports, or other recreational activities?

**6. Empowerment and Political Action**

1. How much control do you feel you have in being involved in making decisions that affect your everyday activities in your neighbourhood? Do you have…

1 No control

2 Control over very few decisions

3 Control over some decisions

4 Control over most decisions

5 Control over all decisions

`

1. Overall, how much impact do you think you have as an individual in making this neighborhood a better place to live?

1 A big impact

2 A small impact

3 No impact

In the past 12 months, have you done any of the following?

1 Yes 2 No

| A. Attend a neighborhood council meeting, public hearing, or public discussion group |  |
| --- | --- |
| B. Met with a politician, called him/her, or sent a letter |  |
| C. Participated in an information or election campaign |  |

1. To what extent do local government and local leaders take into account concerns voiced by you and people like you when they make decisions that affect you?

1 A lot

2 A little

3 Not at all

1. In your opinion, how honest are the officials and staff of the following agencies? Please rate them on a 1 to 5 scale, where 1 is very dishonest and 5 is very honest.

| A. Local government officials |  |
| --- | --- |
| B. Doctors and nurses in health clinic |  |
